# Dietary Pattern-Induced Gut Microbiota Differences Are Associated with White Matter Volume Changes in Middle-Aged Female Macaques

**DOI:** 10.3390/nu18071124

**Published:** 2026-03-31

**Authors:** Brett M. Frye, Haleigh Cooper, Jacob D. Negrey, Courtney Sutphen, Ravinder Nagpal, Jeongchul Kim, Richard A. Barcus, Samuel N. Lockhart, Christopher T. Whitlow, Janet A. Tooze, Hariom Yadav, Suzanne Craft, Thomas C. Register, Carol A. Shively

**Affiliations:** 1 Department of Pathology/Comparative Medicine, Wake Forest University School of Medicine, Medical Center Blvd, Winston-Salem, NC 27157-1040, USA; brett.frye@advocatehealth.org (B.M.F.);; 2 Department of Biology and Biochemistry, Emory and Henry University, 30461 Garnand Drive, Emory, VA 24327-9001, USA; 3 Wake Forest Alzheimer’s Disease Research Center, Wake Forest University School of Medicine, Medical Center Blvd, Winston-Salem, NC 27157-1040, USA; 4 School of Anthropology, The University of Arizona, 1009 E South Campus Dr, Tucson, AZ 85721, USA; 5 Department of Health, Nutrition, and Food Sciences, Florida State University, 120 Convocation Way, Tallahassee, FL 32304, USA; 6 Department of Radiology, Wake Forest University School of Medicine, Medical Center Blvd, Winston-Salem, NC 27157-1040, USA; 7 Department of Internal Medicine/Gerontology, Wake Forest University School of Medicine, Medical Center Blvd, Winston-Salem, NC 27157-1040, USA; 8 Department of Biostatistics and Data Science, Wake Forest University School of Medicine, Medical Center Blvd, Winston-Salem, NC 27157-1040, USA; 9 Department of Neurosurgery and Brain Repair, University of South Florida, 3000 Medical Park Dr #340, Tampa, FL 33613, USA

**Keywords:** *Oscillospira*, Mediterranean diet, metabolomics, insulin resistance, nonhuman primate, *Macaca*, branched chain amino acids, white matter, myelination

## Abstract

**Background/Objectives**: Western and Mediterranean diets have divergent effects on the brain. The gut microbiome may mediate diet effects, and specific microbes may be particularly significant contributors to these processes. *Oscillospira*, a genus of gut-dwelling bacteria, has been implicated as a key microbial target. Other peripheral contributors may include short-chain fatty acids (SCFAs), branched-chain amino acids (BCAAs), insulin resistance, and microbial translocation. **Methods**: We determined the effects of long-term (31 months, ~9 human years) consumption of a Mediterranean or Western-type diet on *Oscillospira* abundance, fecal SCFAs, plasma BCAAs, soluble CD14 (sCD14), and insulin responses in a randomized trial of 38 middle-aged female cynomolgus macaques (*Macaca fascicularis*). We determined diet effects and associations between dependent variables. For variables that were affected by diet composition and significantly associated with *Oscillospira*, we tested whether *Oscillospira* abundance mediated the effects of diet. **Results**: The Mediterranean diet resulted in higher *Oscillospira* (*p* = 0.004) and SCFAs (acetate *p* = 0.002; propionate *p* = 0.049) and lower BCAAs (isoleucine *p* = 0.035; leucine *p* = 0.007; valine *p* < 0.001). The Western diet increased insulin resistance (*p* = 0.040) and WM loss (*p* = 0.011). *Oscillospira* abundance was negatively associated with BCAAs (leucine *p* = 0.007; valine *p* = 0.005) and insulin resistance (insulin AUC: *p* = 0.024; increase in insulin AUC from pretreatment: *p* = 0.020), with trends for isoleucine (*p* = 0.066) and sCD14 (*p* = 0.103). *Oscillospira* abundance was positively associated with acetate (*p* = 0.032) and WM volume changes (*p* = 0.012). *Oscillospira* abundance significantly mediated the effects of diet on white matter volume changes (*p* = 0.020) and on insulin resistance (insulin AUC: *p* = 0.012 at study end; increase in insulin AUC during study: *p* = 0.020), presenting potential pathways through which diet may influence the brain. **Conclusions**: These findings suggest that diet-driven differences in *Oscillospira* are linked to metabolic regulation and white matter integrity, and *Oscillospira* may mediate the relationships. The results highlight a potential role for diet–microbiome interactions in shaping metabolic and brain aging trajectories.

## 1. Introduction

Diet is a promising and practical target for interventions to promote healthy body and brain aging [1]. Adherence to healthier dietary patterns, like the Mediterranean diet, is associated with increased resilience to adverse neurocognitive outcomes, including cognitive impairment and dementia associated with Alzheimer’s disease (AD) [2,3,4,5]. Conversely, unhealthy diets, like the Western-style diet, increase the risk of pathological brain aging [6,7]. The precise mechanisms by which dietary patterns affect the brain remain incompletely understood. Mounting evidence suggests that the influence of diet may be mediated, in part, by the composition of the gut microbiome and its effects on peripheral metabolism. Understanding the relationships between dietary patterns, gut microbial composition, peripheral biomarkers relevant to metabolic health, and the central nervous system (CNS) can provide important insights into the mechanisms linking a healthy diet to neurocognitive health over the life course.

**Figure 1:**
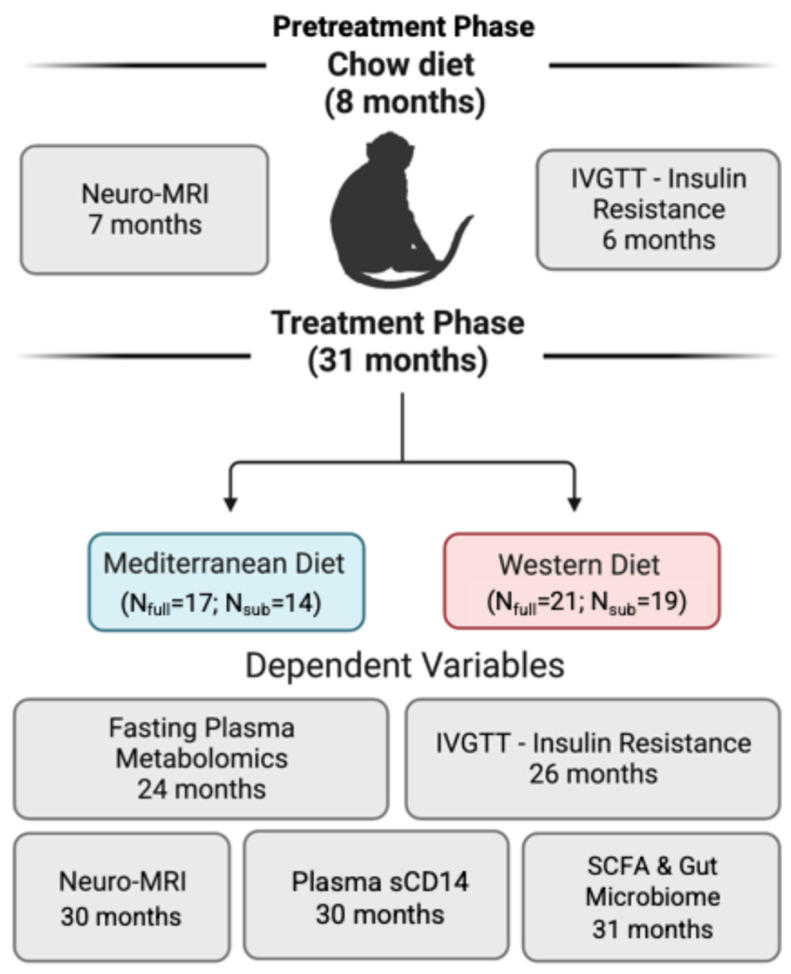
Experimental design of dietary intervention to determine peripheral and central effects of long-term consumption of Mediterranean- vs. Western-style diet in nonhuman primate models of human health and midlife aging.

There may be multiple microbiome-related avenues through which Mediterranean diets may promote healthy body and brain aging (Figure 1). One such avenue is by increasing the prevalence of beneficial microbes, which in turn impact host physiology. Of particular interest is the genus *Oscillospira*, an understudied group of anaerobic gut bacteria (Phylum Firmicutes, Family Ruminococcaceae) [8]. *Oscillospira* abundance is higher in humans with leaner body conditions [9,10,11], and rodent fecal transplant studies suggest that it may stimulate the development of the lean phenotype [12]. *Oscillospira* abundance is sensitive to diet. For example, a high-fat diet in rodents decreased abundance of *Oscillospira*, whereas Mediterranean diet consumption for 1 year increased *Oscillospira* abundance in obese men [13]. Other research indicates both positive and negative associations with various diseases [14], including osteoporosis [15], Parkinson’s disease [16,17], type 2 diabetes [18] autism spectrum disorder [19,20] and chronic systemic inflammation [21]. Given these mixed reports, additional research is warranted to assess health-related effects of *Oscillospira* and factors that impact abundance.

The pathways connecting gut microbes to the brain are an active area of inquiry [1,22,23,24], and relationships between gut microbes and neurocognitive functioning may be due, in part, to their effects on peripheral metabolism [25,26]. Several potential pathways have been proposed. First, the neurological effects of diet may arise, in part, through systemic inflammation and metabolic disturbances. Gut-dwelling microorganisms, such as *Oscillospira*, have the capability to produce metabolites such as short-chain fatty acids (SCFAs) that enhance the integrity of the intestinal barrier, thus restricting microbial translocation and mitigating peripheral inflammation [27,28,29,30]. Growing evidence also suggests that SCFAs positively impact substrate metabolism and function in adipose tissue, skeletal muscle, and the liver, which in turn enhances insulin sensitivity [31]. Conversely, pathogenic microbes, increased with the consumption of Western diets, can compromise the intestinal barrier, resulting in increased microbial translocation across the intestinal barrier [32,33,34].

Circulating branched-chain amino acids (BCAAs; isoleucine, leucine, and valine) are essential amino acids, and they are obtained from the diet. While they are responsive to dietary intervention [35], the gut microbiota acts as a gatekeeper, modulating how much reaches the bloodstream [36]. Disruptions in BCAA metabolism, as evidenced by increased circulating BCAA concentrations, promote obesity and insulin resistance (IR) and predict the onset of type 2 diabetes [37]. Disrupted BCAA metabolism may also promote neurocognitive dysfunction and disease [37] perhaps via IR. IR can disrupt myelination, affecting the oligodendrocytes that create myelin [38], and is linked to lower fractional anisotropy and disrupted white matter (WM) microstructure [39,40]. IR also increases WM hyperintensities which are associated with worsened cognitive function, whereas intranasal insulin reduces WM hyperintensity progression which is associated with improved cognitive function [41]. Thus, diet composition may impact brain structure and function via multiple pathways in the periphery.

In addition, microbial metabolites—including SCFAs—may directly alter the brain’s physiology [42,43]. Of note, *Oscillospira* has been implicated in the direct myelination of white matter tracts [44,45,46,47]. Thus, the relationships between *Oscillospira*, peripheral metabolism, and the brain are poorly understood and require further study.

Nonhuman primate (NHP) models provide important opportunities to study these complex relationships between diet, gut microbes, and host physiology. Like humans, many NHP models—including cynomolgus macaques (*Macaca fascicularis*)—evolved as opportunistic foragers and have a diverse diet consisting of fruits, leaves and other parts of plants, shellfish, crabs, mussels, snails, insects, and fish [48]. Wild macaques often supplement their natural diet by consuming food from human agriculture and garbage [49]. Additionally, their physiological and adverse health responses to Western-like diets mirror those observed in humans [50,51,52,53]. Western-style diets induce coronary and carotid atherosclerosis, obesity, insulin resistance, and type 2 diabetes in these animals [51,52,53,54]. NHPs also share a close phylogenetic relationship to humans, which increases the likelihood of shared biological mechanisms, including those associated with the gut–brain axis. Importantly, NHPs exhibit numerous similarities in neuroanatomy and neurophysiology to humans, making them valuable models for studying brain changes associated with healthy versus pathological aging, including characteristics typical of AD in humans [55,56,57,58,59,60,61,62].

Here, we determined the effects of long-term (31 months, ~9 human years) consumption of a Mediterranean- or Western-style diet on *Oscillospira* abundance, fecal SCFAs, plasma BCAAs, soluble CD14 (sCD14), and insulin responses in a randomized trial of 38 middle-aged female cynomolgus macaques (*Macaca fascicularis*). We targeted this age range, as the etiologies of many chronic conditions (e.g., Alzheimer’s disease [63] and cardiovascular disease [64]) may arise during middle-age. As such, this life stage may be particularly important for understanding mechanisms that promote either risk or resilience to pathological aging later in life. We examined associations between *Oscillospira* abundance and SCFAs, BCAAs, insulin response, soluble cluster of differentiation 14 (sCD14), and white matter (WM) volume change. For variables that were affected by diet composition and significantly associated with *Oscillospira*, we tested whether *Oscillospira* abundance mediated the effects of diet. In a previous, smaller study we showed that the Mediterranean diet induced greater microbial diversity, higher Firmicutes–Bacteroides ratios, and a higher abundance of members of the families Clostridiacea and Lactobacillaceae. The Mediterranean group also had higher abundances of the genera *Lactobacillus*, *Clostridium*, *Faecalibacterium*, and *Oscillospira*, and lower abundances of *Ruminococcus* and *Coprococcus* compared to the Western-style diet group [65]. In addition, compared to those in the Mediterranean-style diet group, subjects consuming the Western-style diet exhibited increased obesity and insulin resistance [51], as well as structural MRI [66] and transcriptional signatures indicative of a neuroinflammatory state [67]. Here, we hypothesized that the Mediterranean-style diet would promote a higher relative abundance of *Oscillospira,* and that *Oscillospira* would be associated with higher levels of SCFAs but lower levels of BCAAs, which promote the development insulin resistance [68]. We expected *Oscillospira* to be associated with lower peripheral inflammatory responses, indicated by the plasma biomarker soluble cluster of differentiation 14 (sCD14) [69]. We also expected higher *Oscillospira* abundances to be associated with CNS profiles indicative of healthy brain aging, including preserved white matter volumes. Finally, we further hypothesized that the relationships between diet, metabolism, and neuroanatomy are mediated by *Oscillospira* abundance, suggesting that *Oscillospira* represents a causal mechanism linking these dietary interventions to their associated health outcomes.

## 2. Materials and Methods

Study Subjects. The overall study sample consisted of thirty-eight middle aged (average starting age = 8.86 years, range = 7.82–10.22 years, age estimated by dentition) female cynomolgus macaques (*Macaca fascicularis*). For most of the analyses (excepting brain volume changes), our sample consisted of N = 33 animals (average starting age = 9.05 years, range = 8.01–10.41 years, age estimated by dentition). The monkeys were purchased from a distributor (SNBL USA SRC, Alice, TX, USA). Following a one-month quarantine, study subjects were housed in small social groups (N = 4–5) in indoor enclosures (3 m × 3 m × 3 m). The groups were maintained on a 12/12 light/dark cycle, with exposure to natural daylight. During the 8-month pretreatment phase, monkeys consumed standard monkey chow (Supplementary Table S1 [51]) with ad libitum access to water. The monkeys were also provided with diverse enrichment—e.g., swimming pools, perches, and manipulation devices—to promote species-typical behavior and ensure psychological well-being. Animals were monitored daily (Monday–Saturday) by research staff. Animal care staff monitored the animals on Sundays. Veterinary care staff were notified immediately with any health-related concerns. The animals established dominance hierarchies during the pretreatment phase, and these hierarchies were stable throughout the experiment. The timing of all experimental protocols is shown in Figure 1 and Supplementary Table S2.

All experimental protocols complied with state and federal regulations and guidelines from the US Department of Health and Human Services. This work was conducted with the approval of the Wake Forest University School of Medicine Institutional Animal Care and Use Committee (IACUC Number: A15-180; approval date 1 May 2016).

Experimental Diets. Following the pretreatment phase, individuals were randomly (using a random number generator) assigned to the Mediterranean-style (N = 17) or Western-style (N = 21) diet groups which were balanced on pretreatment characteristics including body weight, body mass index, circulating basal cortisol, total cholesterol, and triglyceride concentrations [51]. The composition of the experimental diets has been described extensively [50,51,52,54,55,65,70,71,72] and is shown in Supplementary Tables S1 and S3. Briefly, the experimental diets were designed to recapitulate the Mediterranean [73] and Western [74] dietary patterns consumed by humans. While the diets were nearly identical in cholesterol content (~320 mg/2000 kcal/day) and isocaloric in terms of proteins, fats, and carbohydrates, the sources of the ingredients differed. Fats and protein in the Mediterranean-style diet were derived primarily from plant sources, whereas fats and protein in the Western diet were sourced from animal products. Importantly, this difference meant that the diets varied in their ratios of omega-6 to omega-3 fatty acids. The Mediterranean-style diet had a relatively low ratio (approximately 3:1) of these fatty acids, while the Western-style diet had a relatively high ratio (approximately 15:1). Additionally, the Mediterranean-style diet was lower in saturated fats whereas the Western diet was lower in monounsaturated fats.

Feeding Protocol. Each subject was provided with 120 kilocalories of diet per kilogram of body weight each day (120 kcal/kg/day). For a full account of the feeding protocols, see [51,65,70]. During the experiment, the monkeys were trained (on voice command) to enter individual feeding cages located within each social group’s pen. After the subjects consumed the diet, they were released back into their home enclosure.

Fecal Sample Collection. At the end of the experiment (~31 months), the animals were anesthetized with pentobarbital (30–50 mg/kg) to obtain a surgical plane of anesthesia, followed by exsanguination and perfusion with ice-cold saline. This protocol adhered to the guidelines of the American Veterinary Medical Association’s Panel on Euthanasia. Immediately following euthanasia, fecal samples were collected from the rectum into sterile tubes using aseptic techniques and stored at −80 °C.

Microbiome Analysis. See Nagpal et al. [65] for a complete description of the microbiome analysis methods. Genomic DNA was extracted from approximately 200 mg (wet weight) of sample using Qiagen DNA Stool Mini Kits. The V4 hypervariable region of the bacterial 16S rRNA gene was amplified using the primers 515F (barcoded) and 806R, as outlined in the Earth Microbiome Project protocol, with a minor modification (https://earthmicrobiome.ucsd.edu/, accessed on 25 March 2026). The PCR reaction included 25 μL of SYBR R Premix ExTaqTMII, 1 μL of each primer, 5 μL of DNA template, and 18 μL of RNase-free water. The PCR conditions involved an initial step at 50 °C for 2 min, followed by 10 min at 95 °C, and subsequent amplification steps at 95 °C for 30 s, 55 °C for 30 s, and 72 °C for 90 s, for multiple cycles on the Applied Biosystems 7500 Real Time PCR System (Applied Biosystems, Foster City, CA, USA). We stopped the amplification before the fluorescent intensity reached a plateau. The resulting amplicons were purified, quantified, normalized, and pooled together for 16S Miseq analysis. The sample pool was denatured, diluted, and sequenced on an Illumina MiSeq platform using a MiSeq Reagent Kit v3 (Illumina, Inc. San Diego, CA, USA), as previously described [75]. The sequencing process was monitored on the Illumina BaseSpace website using the BaseSpace 16S Metagenomics App. Taxonomic units were assigned using the Quantitative Insights Into Microbial Ecology (QIIME) pipeline and the Greengenes database [76]. The Illumina BaseSpace platform provided demultiplexed sequencing read files, which were quality-filtered, clustered, and analyzed using default parameters in QIIME. After filtering, a total of 1.33 million reads were obtained. Paired-end reads were joined and split using specific scripts. The assembled sequences were grouped into operational taxonomic units (OTUs) at a sequence similarity of 97% identity and were taxonomically classified based on the Greengenes 16S rRNA gene database. Representative OTU sequences were aligned to a Greengenes reference alignment [77], and de novo OTUs were classified using the RDP classifier and the Greengenes reference set with an 80% confidence threshold [78]. Finally, all samples were rarified to an even sequencing depth of 10,000 reads per sample for subsequent analyses.

Statistical Processing of Microbiome Data—After sequence processing in QIIME and rarefaction to an even sequencing depth of 10,000 reads per sample, taxonomic summaries were generated at multiple taxonomic levels. Relative abundance for each taxon was calculated as the proportion of reads assigned to that taxon divided by the total reads per sample following rarefaction. To minimize the influence of sequencing noise and very low-prevalence organisms, analyses were conducted at the genus level and restricted to taxa detected in multiple animals. Taxa that were not consistently detected across samples were excluded from statistical comparisons. Relative abundance values were then used for downstream statistical analyses. Relative abundances of *Oscillospira* were then assessed for normality and homogeneity of variance before parametric analyses were performed.

Metabolomics—Fecal Short-Chain Fatty Acids. We determined the concentrations (µmol/g) of three short chain fatty acids—acetate, propionate, and butyrate—in the end-of-study fecal samples following established protocols [72,79]. Approximately 100 mg of fecal material was suspended in 900 μL of sterile PBS buffer (pH 7.4). Samples were vortexed for 1 min, or until fully homogenized, and then centrifuged at 12,000× *g* for 10 min. The resulting supernatant was collected and passed through a 0.45 μm membrane filter. Concentrations of acetate, propionate, and butyrate (expressed as μmol per gram of feces) were quantified using high-performance liquid chromatography (HPLC) on a Waters 2695 Alliance system (Waters Corporation, Milford, MA, USA) equipped with a diode array detector (DAD) set to 210 nm and an Aminex HPX-87H column (Bio-Rad Laboratories, Hercules, CA, USA). Ten microliters of each sample were injected, and metabolites were separated using 0.005 N H_2_SO_4_ as the mobile phase at a flow rate of 0.6 mL/min with the column maintained at 35 °C.

Metabolomics—Plasma Branched Chain Amino Acids. Branched chain amino acids (BCAAs)—isoleucine, leucine, and valine—were measured in plasma after 24 months of experimental diet consumption. Monkeys were fasted overnight (~18 h), and blood samples were taken the following morning (0700–0800). Blood samples were then centrifuged and plasma-aliquoted according to Metabolon^®^ specifications. The samples were stored at −80 °C until analysis.

Metabolomics analyses were performed by Metabolon (Raleigh, NC, USA). Analyses were conducted using a Waters ACQUITY ultra-performance liquid chromatography (UPLC) system paired with a Thermo Scientific Q-Exactive mass spectrometer (Thermo Fisher Scientific Inc., Waltham, MA, USA). This setup includes a heated electrospray ionization (HESI-II) source and an Orbitrap mass analyzer operating at a mass resolution of 35,000. The scan range spanned from 70 to 1000 *m*/*z*. The raw data first underwent extraction, peak identification, and quality control processing through Metabolon’s proprietary hardware and software. Compound identification was achieved by comparing the results to entries in a library of purified standards or known metabolites. Quantification of peaks was performed using the area-under-the-curve method. The informatics framework comprised a Laboratory Information Management System (LIMS), data extraction and peak identification software, quality control and compound identification tools, and various data interpretation and visualization resources for analysts. Metabolomics data were then normalized to remove platform-specific sources of variability (e.g., systemic error) resulting from inter-day variations in instrument performance. This scaling process allows for metabolite comparisons across batches. Briefly, raw integrated values were calculated for each BCAA using the area under the chromatographic peak. Raw values were then scaled to set the median equal to 1. We report BCAAs as scaled intensity.

Insulin Physiology. We conducted intravenous glucose tolerance tests with insulin responses during the pretreatment phase (at 6 months) and treatment phase (at 26 months) to assess insulin sensitivity. After an overnight (~16–18 h) fast, the monkeys were sedated with ketamine HCl (15 mg/kg), administered dextrose (500 mg/kg), and blood samples were collected at 0, 5, 10, 20, 30, 40, and 60 min post dextrose. Insulin levels were measured using enzyme-linked immunoassay (ELISA; Mercodia, Uppsala, Sweden). We calculated the insulin AUC based on insulin responses observed between 10 and 40 min post dextrose. We calculated the change in insulin AUC by subtracting the pretreatment value from the treatment value. See [51] for a full description of these protocols. Insulin resistance (IR) was inferred from significant increases in insulin AUC between baseline and 26 months, or from significantly greater insulin AUC at 26 months.

Plasma-soluble CD14. At 30 months, we assessed concentrations of the plasma biomarker soluble cluster of differentiation 14 (sCD14) as a marker of immune activation and inflammation frequently associated with gut permeability [34]. Blood was collected during the treatment phase after the animals had consumed experimental diets for 31 months. Prior to sample collection, the animals were fasted overnight for 16–18 h. On the morning of sample collection, individuals were sedated using ketamine hydrochloride (intramuscular, 15 mg/kg ketamine HCl). We collected blood samples via femoral venipuncture, using a 7 mL EDTA tube, which were then placed on ice until subsequent preparation. Plasma samples were then centrifuged for 20 min at 2500 rpm. Plasma was then transferred to Sarstedt tubes (0.2 mL aliquots) and frozen at −80 °C.

Structural Brain Magnetic Resonance Image Acquisition. The methods for image acquisition, processing, and segmentation have been described in detail [55]. We collected structural magnetic resonance images (MRIs) during the pretreatment (7 months) and treatment (~30 months) phases. Prior to MRI, individuals were sedated with ketamine HCl (10–15 mg/kg body weight), followed by anesthesia. Anesthesia was maintained using isoflurane, with 3% for induction and 1.5% for maintenance. T1-weighted anatomic images were obtained utilizing a three-dimensional (3D) volumetric magnetization-prepared rapid acquisition with gradient echo (MPRAGE) sequence. The specific parameters for this sequence were as follows: repetition time (TR) of 2700 ms, echo time (TE) of 3.39 ms, time interval (TI) of 880 ms, flip angle of 8 degrees, 160 slices, and voxel dimension of 0.5 × 0.5 × 0.5 mm^3^. The MRI scans were performed using a 3T Siemens Skyra scanner and a circularly polarized, 32-channel head coil with an internal diameter measuring 18.4 cm (Litzcage, Doty Scientific, Columbia, SC, USA).

Structural MRI—Image Preprocessing. We applied a series of image preprocessing techniques to the data, including bias field correction, and denoising using Advanced Normalization Tools (ANTs) developed by the Penn Image Computing and Science Laboratory at the University of Pennsylvania. We then generated a study-specific template (SST) representing the entire head by utilizing the pretreatment scans through ANTs’ antsMultivariateTemplateConstruction.sh script. The SST was then aligned to the University of North Carolina (UNC) Primate Atlas through rigid-body alignment [80]. The UNC Template brain was registered to the SST brain, and the UNC parcellation map and brain mask were transformed to correspond with our SST using deformable registration (antsRegistrationSyN.sh). The parcellation map encompassed labels including gray matter (GM), white matter (WM), and cerebrospinal fluid (CSF). It also included subcortical structures such as the hippocampus, amygdala, caudate, and putamen, which were not included in this project.

Structural MRI—Longitudinal Image Registration & Segmentation. We performed within-subject registration of the T1-weighted images across the pretreatment and treatment timepoints to estimate longitudinal transformations. We accomplished this via ANTs’ nonlinear registration algorithm (antsRegistrationSyN.sh [81,82]). Next, longitudinal transformations were used to warp the pretreatment brain masks to those of the treatment scans. This method provided individual parcellation maps for brain regions of interest (ROIs) for both time points. Finally, we performed an automatic brain segmentation into cortical (cGM), deep (dGM), and total gray matter (tGM; cortical plus deep GM), WM, and CSF using probabilistic tissue maps from tissue priors generated in SST space. We calculated the percent change in volumes as shown below, which controls for inter-individual differences in monkeys’ head sizes:

$$\frac{\left({Volume}_{exp}-{Volume}_{pre}\right)}{{Volume}_{pre}}\;\times \;100$$

Data Analysis. We first used *t*-tests to determine the effects of diet on the dependent variables: *Oscillospira*, fecal SCFAs (acetate, propionate, and butyrate), circulating BCAAs (isoleucine, leucine, and valine), insulin sensitivity (treatment insulin area under the curve (AUC) and the change in insulin area under the curve from pretreatment (ΔIR)), peripheral inflammation (sCD14), and changes in neuroanatomy. Next, given its potential role as a factor underlying the relationships between diet and body and brain health, we sought to determine the relationships between those dependent variables and *Oscillospira* via Pearson correlations. These analyses were conducted to test a priori hypotheses regarding relationships rather than as a broad exploratory screen. Because the number of comparisons was limited and hypothesis-driven, the results are presented as unadjusted correlations. Accordingly, these associations should be interpreted cautiously and considered hypothesis-generating. For variables that (1) were significantly affected by the experimental diets and (2) significantly associated with *Oscillospira*, we conducted causal mediation analyses. To do this, we first fit a linear regression model testing the relationship between diet (the predictor) and *Oscillospira* abundance (potential mediator). We next used linear regression to test whether the mediator (*Oscillospira* abundance) was significantly associated with the outcome variables. We then conducted causal mediation analyses using the mediate() function from the “mediation” R package (R Package for Causal Mediation Analysis), implementing 500 bootstrap simulations to estimate whether *Oscillospira* abundance mediated the effects of diet on the outcomes of interest. Because the dependent variables were measured using considerably different scales, we scaled the variables using the standardize function in R [83]. Additionally, these analyses should be considered exploratory given the temporal structure of the sampling schedule (Figure 1). All statistical analysis were conducted using GraphPad Prism version 9.5.1 and R version 4.2.2 [84]. For analyses, we assessed normality through visual inspection of data distributions using both histograms and quantile–quantile plots (“qq plots”), which compare observed sample quantiles to those expected under a theoretical normal distribution. When variables appeared to deviate from normality (e.g., skewed distributions), we conducted the corresponding nonparametric analyses. In cases where parametric and nonparametric approaches yielded consistent results, we report the parametric test outcomes for ease of interpretation. We report findings at *p* < 0.10 and determine statistical significance at a 2-sided *p* < 0.05 for all analyses.

## 3. Results

### 3.1. Diet Effects–Peripheral Phenotypes

**Figure 2:**
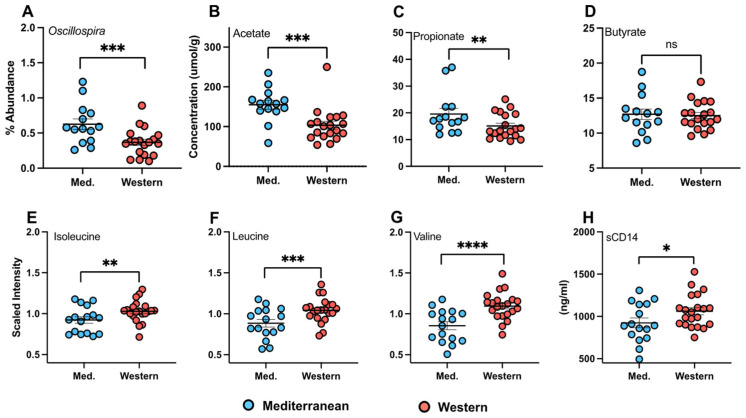
Diet differences in microbial and peripheral phenotypes. Animals consuming the Mediterranean-style diet (N = 14) had higher (**A**) percent abundance of *Oscillospira* (t = 3.068, *df* = 31, *p* = 0.004) and short-chain fatty acids (SCFAs): (**B**) acetate (t = 3.448, *df* = 31, *p* = 0.002), (**C**) propionate (t = 2.052, *df* = 31, *p* = 0.049), but not (**D**) butyrate (t = 0.221, *df* = 31, *p* = 0.826). The Western-style diet group (N = 19) exhibited elevated branched chain amino acids (BCAAs): (**E**) isoleucine (t = 2.198, *df* = 35, *p* = 0.035), (**F**) leucine (t = 2.851, *df* = 35, *p* = 0.007), and (**G**) valine (t = 4.001, *df* = 35, *p* < 0.001); and (**H**) sCD14 (t = 1.196, *df* = 35, *p* = 0.058). “ns” indicates nonsignificant *p* > 0.10, * *p* < 0.10, ** *p* < 0.05, *** *p* < 0.01, and **** *p* < 0.001.

The Mediterranean-style group had higher abundances of *Oscillospira* than the Western-style group (t = 3.068, *df* = 31, *p* = 0.004) (Figure 2A). The fecal SCFAs acetate (t = 3.448, *df* = 31, *p* = 0.002) and propionate (t = 2.052, *df* = 31, *p* = 0.049) were elevated in the Mediterranean group, whereas butyrate did not differ between the diets (t = 0.221, *df* = 31, *p* = 0.826) (N_MED_ = 14; N_WEST_ = 19) (Figure 2B–D). Plasma BCAAs were consistently lower in the Mediterranean-style group than in the Western group: isoleucine (t = 2.198, *df* = 35, *p* = 0.035), leucine (t = 2.851, *df* = 35, *p* = 0.007), and valine (t = 4.001, *df* = 35, *p* < 0.001) (Figure 2E–G). Additionally, as has been previously demonstrated [51], the insulin AUC from the pretreatment to the treatment phase increased significantly in the Western diet group (t = 2.164, *df* = 34, *p* = 0.040). On average, circulating sCD14 tended to be lower in the Mediterranean-style diet group (−115.7 ± 70.55 ng/mL; t = 1.196, *df* = 35, *p* = 0.058) (Figure 2H), suggesting lower gut permeability in those consuming the Mediterranean diet.

### 3.2. Diet Effects–CNS

In agreement with previous reports, the diet groups showed divergent patterns of neuroanatomical change over the course of the experiment. The Western-style diet group was typified by changes in global volumes, whereas the Mediterranean-style diet group was characterized by resilience to change (Supplementary Table S4). Of particular interest for this project, the Western-style group showed losses in bilateral white matter volumes compared to the Mediterranean-style group (t = 2.681, *df* = 36, *p* = 0.011). There were no significant differences for any other neuroanatomic changes (*p* > 0.05).

### 3.3. Peripheral Phenotypes and Oscillospira

**Figure 3:**
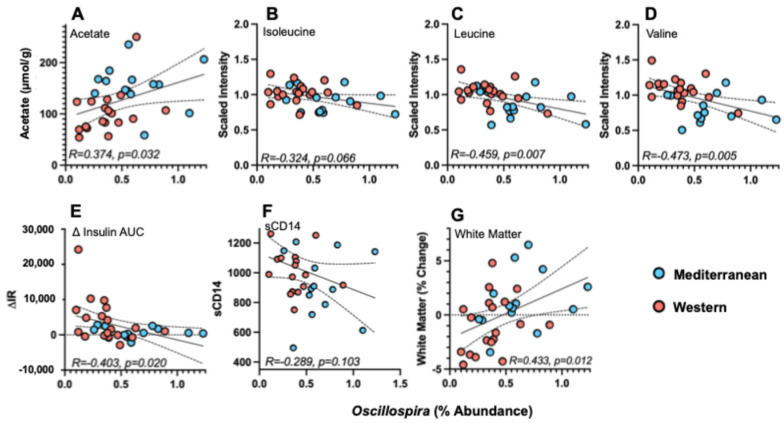
Relationships between experimental phenotypes and *Oscillospira* (N = 33). *Oscillospira* was positively associated with (**A**) the SCFA acetate (R = 0.374, *p* = 0.032). *Oscillospira* was negatively associated with the BCAAs (**B**) isoleucine (R = −0.324, *p =* 0.066), (**C**) leucine (R = −0.459, *p* = 0.007), and (**D**) valine (R = 0.473, *p* = 0.005), as well as (**E**) the change in insulin resistance (ΔIR) over the experiment (R = −0.403, *p* = 0.020) and (**F**) sCD14 (R = −0.289, *p* = 0.103); however, the latter was not statistically significant. (**G**) *Oscillospira* was positively associated with changes in white matter during the experiment (R = 0.433, *p* = 0.012).

*Oscillospira* was significantly correlated with several variables in the periphery (Supplementary Table S5). For SCFAs, *Oscillospira* abundance was positively associated with acetate (R = 0.374, *p* = 0.032; Figure 3A) but not with propionate (R = −0.239, *p* = 0.181) or butyrate (R = −0.097, *p* = 0.590) (Supplementary Table S5). *Oscillospira* was negatively associated with the BCAAs leucine (R =−0.459, *p* = 0.007; Figure 3C) and valine (R = −0.473, *p* = 0.005; Figure 3D). Isoleucine showed a similar negative relationship, although it did not reach statistical significance (isoleucine: R = −0.324, *p* = 0.066; Figure 3B). *Oscillospira* abundance also was negatively associated with insulin AUC (R = −0.393, *p* = 0.024) and changes in insulin AUC from pretreatment (R = −0.403, *p* = 0.020; Figure 3E). One subject in the Western diet group exhibited insulin physiology that may have constituted an outlier data point. However, upon further inspection, the animal’s pretreatment glucose was under 100 mg/dL, indicating that she was successfully fasted prior to the blood draws. She was otherwise unremarkable, with a body condition that fell within the normal distribution of the sample. We therefore re-ran the correlation, omitting this individual, and determined that the relationship between *Oscillospira* and insulin reactivity was still significant for both measures. Finally, *Oscillospira* was negatively associated with sCD14 (R = −0.289, *p* = 0.103; Figure 3F) but the correlation did not reach statistical significance.

### 3.4. Central Phenotypes and Oscillospira

Regarding microbial–CNS relationships, *Oscillospira* abundance was positively associated with the percent change in white matter volumes (R = 0.433, *p* = 0.012; Figure 3G). There were no significant correlations with any other neuroanatomic changes (*p* > 0.091).

### 3.5. Exporatory Mediation of Oscillospira (Figure 4)

**Figure 4:**
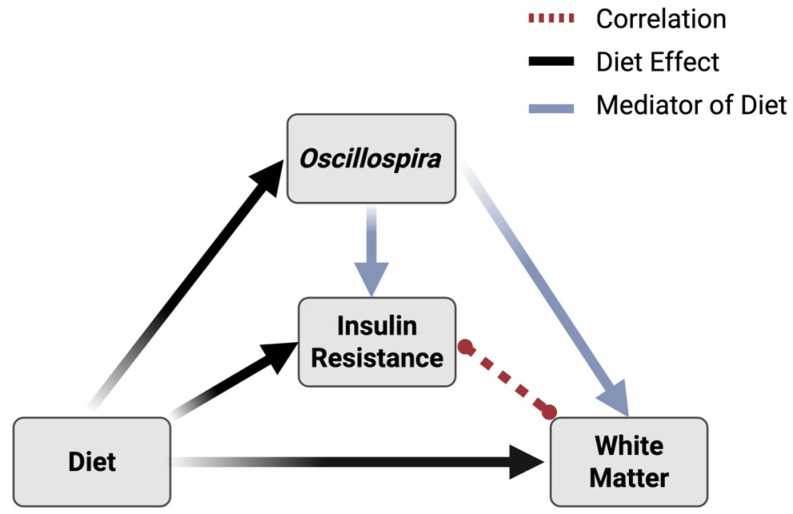
Conceptual representation of effects of *Oscillospira*. *Oscillospira* abundance statistically mediated the relationship between experimental diets and white matter changes and insulin physiology (i.e., delta IR). Worsening insulin resistance, in turn, was correlated with losses in white matter volumes. Correlation shown by dashed red line; main effects of diet shown by black arrows; and mediation effects indicated by blue arrows.

Six dependent variables differed between the experimental diets and were significantly associated with *Oscillospira* abundance: acetate, leucine, valine, insulin AUC, the change in insulin AUC (ΔIR), and white matter volume changes. Of those variables, causal mediation analyses revealed that *Oscillospira* significantly mediated the effects of diet on three variables: the insulin AUC at the experimental time point of 26 months (average causal mediation effect (ACME): β = 0.329, *p* = 0.012), the change from baseline in the insulin response (ACME: β = 0.307, *p* = 0.020), and the percent change in white matter volume (ACME: β = −0.309, *p* = 0.016). The average causal mediation effects of *Oscillospira* for the remaining dependent variables were not statistically significant (Supplementary Table S6). Given these findings, we subsequently tested the hypothesis that IR may, in turn, directly impact white matter. To test this hypothesis, we determined the correlations between the two measures of IR and white matter changes over the experimental period. Experimental insulin AUC was associated with white matter changes; however, this relationship did not reach statistical significance (R = −0.334; *p* = 0.058). The relationship between WM and insulin AUC change from baseline (ΔIR) was statistically significant, with higher IR being associated with greater losses of cerebral white matter (R = −0.395; *p* = 0.023). A working exploratory model of the relationships between diet, *Oscillospria* abundance, IR and white matter changes is shown in Figure 4.

## 4. Discussion

The data reported here derive from a randomized preclinical NHP trial in which diet treatment groups were balanced on pretreatment characteristics, thus allowing for causal inferences with respect to diet effects. Diets were designed to emulate diet patterns currently consumed by humans. We chose to study diet patterns rather than individual nutrients because of the complex interaction of all diet components with the gut microbiome. The trial design, diet compositions, and use of NHPs increase the likelihood of accurate translation of these findings to human health. Diet represents an effective and practical target to promote healthy aging trajectories, and such interventions may target microbiome–host interactions to promote metabolic and brain health.

**Figure 5:**
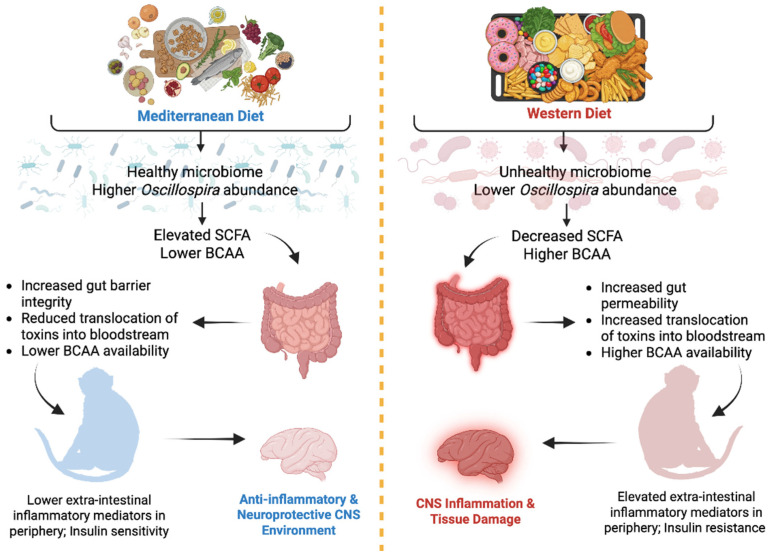
Working model of microbiome-host interactions impacting metabolic and brain health in this intervention study of Mediterranean- and Western-style diets, illustrating potential pathways linking dietary patterns to peripheral and central processes that promote risk or resilience to pathological aging.

Congruent with a small body of clinical evidence linking *Oscillospira* with Mediterranean diet consumption [13], the present study demonstrates that consumption of a Mediterranean-style diet resulted in higher abundances of *Oscillospira* and better gut barrier integrity compared to the Western-style diet. Consumption of a Mediterranean-style versus Western-style diet also resulted in favorable peripheral metabolic profiles and neuroanatomical preservation. Specifically, Mediterranean-style diet-fed animals exhibited higher levels of beneficial microbial metabolites (SCFAs), lower circulating BCAAs, and attenuated the aging-related increase in IR over time. These findings were accompanied by greater white matter preservation, emphasizing the broad, systemic benefits of Mediterranean-style diet on aging-related outcomes. *Oscillospira* abundance was correlated with many of these health-related phenotypes. Furthermore, mediation analyses suggested that the relationships between diet, IR, and neuroanatomy were mediated by *Oscillospira* abundance, suggesting that *Oscillospira* represents an underlying mechanism linking Mediterranean and Western diet patterns to these health outcomes. These pathways also may involve direct relationships between IR and cerebral white matter. Collectively, these findings suggest that adherence to a Mediterranean diet may enhance microbial environments that confer healthy host phenotypes across multiple, interrelated systems (Figure 5).

Previous work showed that the Mediterranean diet increased several microbial genera that produce SCFAs, including *Lactobacillus*, *Clostridium*, *Faecalibacterium*, and *Oscillospira* [65]. Increasing the levels of SCFAs such as acetate, lactate, and butyrate may promote healthy aging outcomes by improving the barrier integrity of the gut epithelium, thereby reducing the translocation of endotoxins into host interstitial and circulating tissues [14,85,86,87]. Reducing endotoxin translocation, in turn, may dampen peripheral inflammatory responses. Consistent with this hypothesis, individuals in the Mediterranean diet group tended to exhibit lower levels of circulating sCD14, an inflammatory biomarker released by macrophages in response to endotoxin exposure [34]. Thus, the Mediterranean diet likely improved gut barrier integrity, dampening peripheral inflammatory responses originating in the gut.

*Oscillospira* is a genus of interest for probiotic interventions on inflammatory conditions and metabolic disease [14]. This interest is supported by the clinical observation that *Oscillospira* abundance was negatively associated with pro-inflammatory monocyte chemoattractant protein-1 (MCP-1) [21].

Alternatively, SCFAs may directly impact CNS physiology. In the CNS, SCFAs play key roles in neurodevelopment, the regulation of neuroinflammatory processes, the maturation of microglia, adult neurogenesis, the modulation of neurotransmitter and neurotrophic factor production, and the consolidation of memory [88]. Acetate, specifically, may play a role in oligodendrocyte functioning and resulting white matter integrity. Acetate has been shown to epigenetically regulate gene expression in oligodendrocytes through histone acetylation and methylation—key epigenetic mechanisms that influence cellular differentiation, including the maturation of oligodendrocytes [89,90].

The gut microbiota is implicated in increasing circulating BCAAs via de novo synthesis [36,91]. Compared to the Mediterranean group, monkeys consuming the Western-style diet had higher BCAAs, which may act as signaling molecules to promote peripheral inflammation [92]. In vitro studies have shown that elevated BCAAs may promote oxidative stress and pro-inflammatory states in peripheral blood mononuclear cells via the activation of the transcription factor NF-κB, a central regulatory mediator of inflammation which promotes the expression of cytokines and adhesion molecules [92]. Our previous work demonstrated that the Western diet group had elevated pro-inflammatory transcriptional profiles including elevations in IL6, IL1α, NF-κB1, and NF-κB2 transcripts compared to the Mediterranean group [93].

Elevated BCAAs due to Westen-style diet consumption may also perturb peripheral metabolism via the development of IR. Both preclinical [94] and clinical [95] studies have demonstrated that elevated BCAAs promote IR. The mechanisms underlying this relationship may include disrupted insulin signaling in skeletal muscle [96]. Consistent with the hypothesis that minimizing BCAAs may protect from developing IR, animals consuming the Mediterranean-style diet exhibited an attenuated increase in insulin AUC over time relative to the Western group. Thus, Mediterranean diets may protect metabolic health during aging, in part, via mitigation of BCAA-associated activity.

Taken together, our results suggest that the Mediterranean-style diet, rich in plant proteins and fiber, promoted the growth of SCFA-producing microbes, whereas the Western-style diet, rich in animal fats and proteins, supported the growth of microbes that produce higher proportions of BCAAs. Such a metabolic shift in the gut microbiome may contribute to the observed differences in the fecal and circulating metabolic profiles, and the health-related sequelae observed in this trial.

In concordance with clinical observations, *Oscillospira* abundance was positively associated with white matter volume preservation and appeared to mediate this relationship in our models. However, *Oscillospira* showed no other neuroanatomical correlations. These results suggest a potential link between *Oscillospira* abundance and pathways related to oligodendrocyte functioning and myelination, although experimental intervention is needed to establish causality. One possible mechanism involves SCFAs, which are produced by *Oscillospira* and other microbes enriched by the Mediterranean diet. Preclinical experiments have shown that SCFA administration can suppress demyelination and promote remyelination through oligodendrocyte maturation and differentiation [97]. This specific link highlights the need to explore white matter as a sensitive neural target for microbiome–diet interactions. Alternatively, the relationships between gut microbiota including *Oscillospira* and neuroanatomy may be indirect. Indeed, our results suggest that *Oscillospira* mediated the impact of diet on IR development, which, in turn, was correlated with changes in white matter volumes. While these findings are biologically plausible, causality cannot be determined in this trial. Future studies should aim to elucidate the underlying mechanisms of the associations between white matter, IR, and SCFA-producing microbiota, including diffusion-weighted and diffusion tensor imaging, to inform WM microstructural integrity.

This study has some limitations. First, although we showed that diet composition altered microbiome composition and neural outcomes, several mechanistic pathways that may relate the two remain unexplored. Specifically, gut microbes are known to produce neuroactive compounds (e.g., serotonin, dopamine, γ-aminobutyric acid, norepinephrine) that can influence both enteric and CNS function, which were not assessed in the current study. Indeed, future work should aim to determine the functional implications of these dietary patterns on the central nervous system through analyses such as diffusion tensor and diffusion-weighted neuroimaging. Endocrine mechanisms also potentially mediate microbiota–brain interactions, but these factors were not evaluated here. While *Oscillospira* has been previously associated with social behavior, this relationship was not examined in the present study. Relatedly, future studies should aim to investigate the interactions between other factors such as the social environment—including the role of psychosocial stress—and diet. Sex-specific effects and menstrual cycle variation, which may have increased variability in the data, could not be evaluated. Longitudinal data of the microbiome, with temporally separated mediator and outcome measures, would considerably strengthen this body of work, especially considering the broader limitations of microbiome manipulation studies and the mixed evidence regarding persistent microbial shifts [98]. Advances in technology (e.g., ASV-based pipelines and updated reference databases) will help refine the taxonomic resolution, increasing certainty of genus-level assignments as well as making identification of species-level targets possible. Important to note, the statistical analyses were performed to generate hypotheses for future testing and do not imply causality.

## 5. Conclusions

Taken together, these findings suggest that dietary modulation of the gut microbiome—particularly enrichment of taxa like *Oscillospira*—may influence aging trajectories through coordinated effects on metabolic regulation and brain structure, and add to the growing literature supporting the health benefits of the Mediterranean diet [2,5,50,55,66,67,99,100,101,102,103,104,105,106]. This work highlights the complexity of relationships between diet, the gut microbiome, systemic physiology, and neurobiology. Further experiments are needed in which *Oscillospira* levels are manipulated and functional outcomes including cognitive function are measured. As precision nutrition strategies gain traction in geroscience, our findings underscore the promise of leveraging diet–microbiome interactions to promote healthy aging.

## Data Availability

The data are available upon request at www.WakeShare.org.

## Supplementary Materials

The following supporting information can be downloaded at: https://www.mdpi.com/article/10.3390/nu18071124/s1, Supplementary Table S1. Composition of experimental diets; Supplementary Table S2. Experimental design, including timeline of variables and sample sizes of each assay; Supplementary Table S3. Recipes of experimental diets; Supplementary Table S4. Differences across diet groups in global neuroanatomical changes over the course of the experiment (31 months); Supplementary Table S5. Correlations between *Oscillospira* and dependent variables in the periphery and the CNS (N = 33); Supplementary Table S6. Exploratory mediation analysis assessing how *Oscillospira* may mediate the effects of diet (N = 33) [51,74,107,108,109,110,111,112].

## Abbreviations

The following abbreviations are used in this manuscript:

BCAA	Branched Chain Amino Acid
SCFA	Short-Chain Fatty Acid
CNS	Central Nervous System
IR	Insulin Resistance
AUC	Area Under the Curve
NHP	Nonhuman Primate
ROI	Regions of Interest
cGM	Cortical Gray Matter
dGM	Deep Gray Matter
tGM	Total Gray Matter
WM	White Matter
CSF	Cerebrospinal Fluid
