# Supplementary material for: Dietary Pattern-Induced Gut Microbiota Differences Are Associated with White Matter Volume Changes in Middle-Aged Female Macaques
Source: Nutrients. 2026 Mar 31;18(7):1124. doi: 10.3390/nu18071124 (PMC13075110; doi:10.3390/nu18071124)
Supplement: Supplementary file 1 [file nutrients-18-01124-s001.zip › nutrients-4133816-supplementary.pdf]

nutrients

MDPI

Article

Dietary Pattern-Induced Gut Microbiota Differences Are Associated with White Matter Volume Changes in Middle-Aged Female Macaques

Brett M. Frye [1,2,3], Haleigh Cooper [1,2], Jacob D. Negrey [1,4], Courtney Sutphen [1,3], Ravinder Nagpal [5], Jeongchul Kim [3,6], Richard A. Barcus [3,6], Samuel N. Lockhart [3,7], Christopher T. Whitlow [3,6], Janet A. Tooze [8], Hariom Yadav [9], Suzanne Craft [3,7], Thomas C. Register [1,3] and Carol A. Shively [1,3,*]

1 Department of Pathology/Comparative Medicine, Wake Forest University School of Medicine, Medical Center Blvd, Winston-Salem, NC 27157-1040, USA; brett.frye@advocatehealth.org (B.M.F.)
2 Department of Biology and Biochemistry, Emory and Henry University, 30461 Garnand Drive, Emory, VA, 24327-9001, USA
3 Wake Forest Alzheimer's Disease Research Center, Wake Forest University School of Medicine, Medical Center Blvd, Winston-Salem, NC 27157-1040, USA
4 School of Anthropology, The University of Arizona, 1009 E South Campus Dr, Tucson, AZ 85721, USA
5 Department of Health, Nutrition, and Food Sciences, Florida State University, 120 Convocation Way, Tallahassee, FL 32304, USA
6 Department of Radiology, Wake Forest University School of Medicine, Medical Center Blvd, Winston-Salem, NC 27157-1040, USA
7 Department of Internal Medicine/Gerontology, Wake Forest University School of Medicine, Medical Center Blvd, Winston-Salem, NC 27157-1040, USA
8 Department of Biostatistics and Data Science, Wake Forest University School of Medicine, Medical Center Blvd, Winston-Salem, NC 27157-1040, USA
9 Department of Neurosurgery and Brain Repair, University of South Florida, 3000 Medical Park Dr #340, Tampa, FL 33613, USA; hyadav@usf.edu

Supplementary Information

Supplementary Table S1. Composition of experimental diets.

	Human		Nonhuman Primate		
	Western	Mediterranean	Western*	Mediterranean*	Chow#
% of Calories					
Protein	15[a]	17[b]	16	16	18
Carbohydrate†	51[a]	51[b]	54	54	69
Fat	33[a]	32[b]	31	31	13
% of Total Fats					
Saturated	33[a]	21[b]	36	21	26
Monounsaturated	36[a]	56[b]	36	57	28
Polyunsaturated	24[a]	15[b]	26	20	32
Other Nutrients					
ω6:ω3 Fatty Acids	15:1[c]	2.1-3:1[d]	14.8:1	2.9:1	12:01
Cholesterol mg/Cal	0.13[a]	0.16[b]	0.16	0.15	trace
Fiber g/Cal	0.01[a]	0.03[e]	0.02	0.04	0.01
Sodium mg/Cal	1.7[a,f]	1.3[b,e]	1.7	1.1	0.25

* Developed and prepared at Wake Forest School of Medicine (Shively et al. 2019) [51]

# LabDiet Chemical Composition Diet 5037/8. Type of fat known in 86% of total fat. Omega-6 from corn and pork fat.

† Human carbohydrate calories include alcohol.

[a] (US Department of Agriculture, 2016) [74]

[b] (Bédard et al., 2012) [107]

[c] (Simopoulos, 2006) [108]

[d] (Cordain et al., 2005) [109]

[e] (Kafatos et al., 2000) [110]

[f] (Powles et al., 2013) [111]

reprinted with permission (Shively et al., 2019) [51]

Supplementary Table S2. Experimental design, including timeline of variables and sample sizes of each assay.

Experimental Variable	Variable Description	Timing of Collection	N
Insulin AUC	insulin AUC based on insulin responses observed between 10- and 40-minutes post dextrose during IVGTT	Pretreatment Phase (at 6 months)<br>Treatment phase (at 26 months)	N_{PRE}=33<br>N_{TREAT}=33
Change in insulin AUC (Delta Insulin Resistance (ΔIR)	change in insulin AUC (Pretreatment subtracted from the treatment value)	Pretreatment Phase (at 6 months)<br>Treatment phase (at 26 months)	N=33
Structural MRI (Brain Volumes; Percent changes)	Percent changes from pretreatment to treatment phase	Pretreatment Phase (at 7 months)	N_{PRE}=38<br>N_{TREAT}=38

		Treatment phase (at 30 months)	
Branched Chain Amino Acids (BCAAs)	BCAA in plasma (scaled intensity)	Treatment Phase (at 24 months)	N=33
Short Chain Fatty Acids (SCFAs)	Fecal SCFA (μmol/g)	Treatment Phase (at 31 months)	N=33
soluble CD14 (sCD14)	sCD14 in plasma (ng/ml)	Treatment Phase (at 30 months)	N=33
Oscillospira	Fecal *Oscillospira* (percent abundance)	Treatment Phase (at 31 months)	N=33

Supplementary Table S3. Recipes of experimental diets.

**Western**		**Mediterranean**	
Ingredient	g/kg	Ingredient	g/kg
Casein, USP	85.0	Casein, USP	17.4
Whey protein, 895	85.0	Whey protein, 895	17.4
		Dried egg white	26.1
		Fish meal (menhaden)	26.1
		English walnut powder	8.7
		Black bean flour	43.5
		Garbanzo bean flour	17.4
		Wheat flour (all-purpose)	243.5
Dextrin	260.0	Dextrin	96.6
Sucrose	180.0	Sucrose	34.8
High fructose corn syrup, 55	70.0	Banana	130.4
		Applesauce	38.2
		Tomato paste	17.4
Cellulose (Alpha-Cel)*	79.4	Cellulose (Alpha-Cel)*	94.8
Lard	41.5	Olive oil (Filippo Berio Extra-Virgin)	61.7
Beef tallow HHR	40.0	Menhaden oil (OmegaPure)	8.7
Butter, lightly salted	12.5	Butter, lightly salted	8.7
Corn oil	35.0	Corn oil	10.4
Flaxseed oil	3.0	Flaxseed oil	1.7
Dried egg yolk	6.0	Dried egg yolk	14.8
Crystalline cholesterol	0.4		
Complete vitamin mix (Teklad)	25.0	Complete vitamin mix (Teklad)	21.7
Mineral Mix w/o Ca, P, NaCl	50.0	Mineral mix w/o Ca, P, NaCl	43.5
Calcium carbonate	4.3	Calcium carbonate	3.7
Calcium phosphate, monobasic	7.5	Calcium phosphate, monobasic	6.5
NaCl (table salt)	16.0	NaCl (table salt)	6.3
Total	1,000	Total	1,000

* Total fiber (percent of diet): WEST, 7.94; MED, 12.7.
Reprinted from Shively et al. 2019 with permission [51].

Supplementary Table S4. Differences across diet groups in global neuroanatomical changes over the course of the experiment (31 months). Statistical differences assessed via unpaired t tests (N=38).

Global Brain Volume (mm^3)	Average Change – Mediterranean Diet	Average Change – Western Diet	t value	p value
Total Brain Volume	0.129	2.307	3.898	**<0.001**
Total Gray Matter	-0.522	3.536	3.608	**0.001**
Cortical Gray Matter	-0.487	5.222	3.276	**<0.002**
AD-Meta ROI	0.012	10.610	3.651	**0.001**
White Matter	1.474	-0.827	2.681	**0.011**
Cerebrospinal Fluid	1.551	-5.233	2.757	**0.009**

Supplementary Table S5. Correlations between *Oscillospira* and dependent variables in the periphery and the CNS (N=33). Significant values (p<0.05) are shown in **bold,** and those correlations p<0.10 are *italicized*.

Variable	Variable Type	Pearson Correlation Co-efficient	p value
Peripheral Variables			
Acetate	SCFA	0.374	**0.032**
Propionate	SCFA	-0.239	0.181
Butyrate	SCFA	-0.097	0.590
Isoleucine	BCAA	-0.324	*0.066*
Leucine	BCAA	-0.459	**0.007**
Valine	BCAA	-0.473	**0.005**
Insulin AUC	Insulin Physiology	-0.393	**0.024**
Delta IR	Insulin Physiology	-0.403	**0.020**
sCD14	Inflammation	-0.289	0.103
CNS Variables			
Total Brain Volume (% change)	Neuroanatomy	-0.215	0.229
Total Gray Matter (% change)	Neuroanatomy	-0.299	*0.091*
Cortical Gray Matter (% change)	Neuroanatomy	-0.269	0.131
AD Meta ROI (% change)	Neuroanatomy	-0.288	0.104
White Matter (% change)	Neuroanatomy	0.433	**0.012**
Cerebrospinal Fluid (% change)	Neuroanatomy	0.144	0.423

Supplementary Table S6. Exploratory mediation analysis assessing how *Oscillospira* may mediate the effects of diet (N=33). Only dependent variables significantly associated with *Oscillospira* (p < 0.05; Supplementary Table 3) were included. The Average Causal Mediation Effect (ACME) quantifies the effect of the treatment ("Diet") on the outcome variables through the mediator (*Oscillospira* abundance). The Average Direct Effect (ADE) represents the effect of diet

after accounting for the mediator. The Total Effect reflects the influence of the experimental diet on each outcome variable without accounting for the mediator. The Proportion Mediated indicates the percentage of the total effect attributable to mediation. Model estimates and p-values for each variable are provided. The mediation models used bootstrapping with 500 simulations to determine statistical significance (Tingley et al. 2014) [112].

	β Estimate	p value
Acetate		
ACME	-0.151	0.412
ADE	-0.898	0.036
Total Effect	-1.049	<0.001
Proportion Mediated	0.144	0.412
Leucine		
ACME	0.308	0.16
ADE	0.568	0.08
Total Effect	0.877	<0.001
Proportion Mediated	0.352	0.16
Valine		
ACME	0.243	0.172
ADE	0.907	0.012
Total Effect	1.151	<0.001
Proportion Mediated	0.211	0.172
Insulin AUC at Time 26mo		
ACME	0.329	0.012
ADE	0.208	0.324
Total Effect	0.537	0.032
Proportion Mediated	0.612	0.036
Delta IR		
ACME	0.307	0.020
ADE	0.345	0.120
Total Effect	0.653	0.004
Proportion Mediated	0.471	0.016
White Matter (% Change)		
ACME	-0.309	0.016
ADE	-0.464	0.144
Total Effect	-0.772	0.012
Proportion Mediated	0.400	0.028
